# Analysis of compliance and language use in broad informed consent forms in international multicenter clinical trials after the implementation of the 2018 common rule

**DOI:** 10.1186/s12910-026-01483-7

**Published:** 2026-05-20

**Authors:** Pannita Anek, Nahathai Dukaew, Nimit Morakote, Nattharinee Kongta, Mingkwan Na Takuathung, Nut Koonrungsesomboon

**Affiliations:** 1 Department of Pharmacology, Faculty of Medicine, Chiang Mai University, Chiang Mai, 50200 Thailand; 2 Clinical Research Center for Food and Herbal Product Trials and Development (CR-FAH), Faculty of Medicine, Chiang Mai University, Chiang Mai, 50200 Thailand; 3 School of Health Science, Mae Fah Luang University, Chiang Rai, 57100 Thailand; 4 Department of Parasitology, Faculty of Medicine, Chiang Mai University, Chiang Mai, 50200 Thailand

**Keywords:** Broad consent, Informed consent forms, Clinical trials, Readability, Biospecimens, Ethical guidelines

## Abstract

Broad consent refers to a consent model that permits the collection, storage, and future use of participants’ biospecimens and related data under ethical oversight. Broad informed consent forms (ICFs) are standalone documents used to operationalize this model by recording participants’ permission for such secondary use. Although the 2018 Revised Common Rule and the 2016 CIOMS Guidelines specify the essential elements required in broad ICFs, systematic evaluations of compliance and readability in international multicenter clinical trials remain limited. This study analyzed 54 broad ICFs used between 2019 and 2024 in international multicenter clinical trials involving a research site at the Faculty of Medicine, Chiang Mai University, Thailand. Compliance with ethical and regulatory requirements for essential elements of broad consent was evaluated, and readability of English-language ICFs was assessed using the Flesch Reading Ease (FRE) and Flesch-Kincaid Grade Level (FKGL) metrics. Most ICFs included over 90% of essential elements, with high compliance in consent documentation (100%, *n* = 54), storage conditions and duration (98.2%, *n* = 53), and voluntary participation (98.2%, *n* = 53). However, some elements were frequently incomplete or absent, including explanations of the meaning of broad consent (64.8%, *n* = 35) and information on commercial profit sharing (51.9%, *n* = 28). Readability analyses posed additional challenges: average FRE indicated difficult text (55.1 ± 7.0; Median 55.6; IQR 6.8), and mean FKGL exceeded recommended levels for health communication (9.7 ± 1.5; Median 9.7; IQR 1.9). Thai-language versions were consistently longer than English version in both word count (3,020.2 ± 1,442.1 vs. 2,188.0 ± 1,128.4 words) and page length (9.2 ± 3.8 vs. 7.1 ± 2.8 pages). Overall, while most broad ICFs meet relevant regulatory requirement, variation in content completeness, considerable length and limited readability may reduce their effectiveness in supporting participant understanding in research involving the long-term storage and future use of biospecimens. This study addresses an important gap by examining the actual content and readability of broad ICFs used in international multicenter clinical trials in Thailand.

## Introduction

Broad consent has increasingly been adopted as an alternative to study-specific consent processes for obtaining permission from participants for the collection, storage, and future use of their biospecimens and related data. In contrast to study-specific consent, which limits biospecimen use to a defined research project, broad consent allows for the future research use of biospecimens and/or related data to a broader scope, under appropriate governance and ethical oversight. Proponents of broad consent argue that this approach provides flexibility for studies involving biospecimens while ensuring that participants receive sufficient information about the scope, risks, and management of such use to make an informed decision [1]. However, critics have raised concerns that broad consent may be insufficient to provide adequate detail regarding future research directions, potential risks, and data management procedures [2].

To ensure that broad consent is ethically and legally acceptable, ethicists and relevant policy and regulatory bodies have developed a set of elements to be included in broad informed consent forms (ICFs), the document used to obtain permission for the collection, storage, and future use of biospecimens and/or related data. In recognition of the growing importance of broad consent, the Council for International Organizations of Medical Sciences [3] provides detailed ethical issues and considerations for the collection, storage, and use of biospecimens and/or related data in the International Ethical Guidelines for Health-related Research Involving Humans [3]. A few years later, the 2018 revision to the U.S. Federal Policy for the Protection of Human Subjects (the “Common Rule”) established regulatory requirements in federally funded research and delineate essential elements of broad consent [4].

Despite ethical and regulatory advancements in the last decade, systematic evaluation of broad ICFs remains limited. Two major aspects warrant particular attention: regulatory compliance and readability. First, regarding compliance, few studies have examined whether broad ICFs adequately meet the internationally recognized ethical and regulatory elements applicable to multinational clinical research. Previous ethical analyses have reported incomplete inclusion of essential elements, inconsistent presentation of information, and variation in the clarity of key disclosures across institutions [5, 6]. Consequently, participants may provide broad consent without fully understanding its meaning, associated risks, and other essential information, often due to unclear or insufficient explanations [7]. Assessing the extent to which current broad ICFs comply with the required elements is therefore critical to identify areas of deficiency and ensure ethical conduct of research involving biospecimens [8].

Second, in terms of participant understanding, broad consent presents challenges related to the complexity of the information and the contexts in which consent is sought, which may hinder understanding of how biospecimens and related data will be used [9]. Digital platforms and alternative consent models have been proposed to improve information delivery and participant engagement [10, 11]. However, explaining the broad and future use of data remains difficult. In addition, studies show that consent documents in general are often lengthy and have low readability [12]. Given the additional conceptual demands of broad consent, clear communication is essential for informed decision-making [13].

A comprehensive assessment of both ethical and regulatory compliance and readability of broad ICFs is therefore essential to identify deficiencies, promote good practices, and strengthen participant protection in research involving biospecimens. The present empirical study, therefore, aimed (1) to describe the characteristics and compliance of broad ICFs used in international multicenter clinical trials involving the collection, storage, and potential future use of biospecimens, and (2) to determine the readability level of the written language used in these documents.

## Materials and methods

### Study design and eligibility criteria

This study was an empirical cross-sectional study evaluating the compliance and language readability of broad ICFs. The study protocol received the certificate of exemption from ethical review from the Research Ethics Committee (REC) of the Faculty of Medicine, Chiang Mai University, Thailand (Certificate No. EXEMPTION 8976/2565). Relevant broad ICFs were retrieved from the institutional database by authorized REC personnel at the request of the investigators, with all data handled confidentially. Only ICF versions that had received formal approval from the REC of the Faculty of Medicine, Chiang Mai University, Thailand, were included in the analysis to ensure that the evaluation reflected documents authorized for actual use with participants.

Broad ICFs were included if they met the following criteria: (1) the form was used in a international multicenter clinical trial involving the collection, storage, and/or use of biospecimens, with the study protocol and related documents (including broad ICFs) reviewed and approved by the REC of the Faculty of Medicine, Chiang Mai University, between January 2019 and December 2024; (2) the broad ICF was available in both English and Thai; and (3) the broad ICF was designed for potential research participants capable of providing informed consent. Two reviewers independently screened the documents for inclusion, and any disagreements were resolved through discussion and consensus with the third reviewer.

### Data collection and extraction framework

The data extraction form was developed based on the essential elements for broad consent, as outlined in the 2018 Revised Common Rule and the 2016 CIOMS Guidelines, by modifying the form used in our previous study [14]. These frameworks were selected because they provide internationally recognized standards applicable to international multicenter trials.

Data were extracted from both the English and Thai versions of the broad ICFs across the following three domains: (1) study characteristics (i.e., study title, sponsor name and origin, trial phase, trial design, disease or condition being studied (classified according to ICD-10), number of research participants, type of optional future research using biospecimens, which was recorded and categorized into human genetic, or non-genetic biomarker, and the biospecimen collection method; (2) broad ICF characteristics (i.e., scope of the broad ICF, the title or heading of the form, document length (number of pages), and total word count). These were standalone broad ICFs, provided separately from the main clinical trial consent forms; and (3) elements of broad consent and language use within each element. The presence and description of 15 essential elements were evaluated using a directed content analysis approach [15]. Consistent with this method, coding categories were predefined based on the 2018 Revised Common Rule and the 2016 CIOMS guidelines to systematically analyze the ICFs. These elements were grouped into four categories: general content, biospecimen-specific issues, risk-benefit disclosures, and consent documentation (see Table 1 for a complete list of elements). Each element was assessed and categorized as: (1) completely provided (2), incompletely or incorrectly provided (3), not provided, or (4) not applicable. Specifically, elements were categorized as not provided (absent) when entirely missing and incompletely provided (inadequate) when the description lacked essential details or relied solely on cross-referencing the main study’s PICF without providing sufficient information within the standalone document. Assessment was independently conducted by two reviewers, with discrepancies resolved by consensus or through consultation with a third reviewer.

**Table 1:** Broad ICF elements required in the two major ethical guidelines and regulations

ICF elements	Revised Common Rule (2018)	CIOMS (2016)	Explanation of what information is expected to be provided in a broad ICF
1. General items			
1.1 Meaning of broad consent	x		A statement that the participant will not be informed of the details of any specific research studies that might be conducted using his/her biospecimens, including the purposes of the research
1.2 Purpose of the biobank and rules of access		x	Purpose of the biobank; the rules of access to the biobank
1.3 Voluntary participation	x		That participation is voluntary (i.e., the right to refuse and the right to withdraw consent without penalty or loss of benefits to which the participant is otherwise entitled)
1.4 Confidentiality	x		Confidentiality of records
1.5 Relevant results to be disclosed	x	x	A statement that clinically relevant research results may not be disclosed to the participant unless it is known that such results, including individual research results, will be disclosed to the participant in all circumstances; the possibility of unsolicited findings, and how they will be dealt with
1.6 Contact information	x	x	An explanation of whom to contact for answers to questions about the participant’s rights and about storage and use of his/her biospecimens, and whom to contact in the event of research-related harm; how the participant can contact the biobank custodian
2. Items related to biospecimens			
2.1 Type of biospecimens	x		Biospecimens that might be used in research
2.2 Type of research	x	x	Foreseeable uses of the biospecimens, whether limited to an already fully defined study or extending to a number of wholly or partially undefined studies; types of research that may be conducted with biospecimens, including whether the research will (if known) or might include whole-genome/exome sequencing (i.e., sequencing of a human germline or somatic specimen with the intent to generate the genome or exome sequence of that specimen)
2.3 Intended goal of the use of biospecimens		x	The intended goal of the use of biospecimens, whether only for basic or applied research, or also for commercial purposes
2.4 Commercial profit sharing	x		A statement that the participant’s biospecimens (even if identifiers are removed) may be used for commercial profit, and whether the participant will or will not share in this commercial profit
2.5 Type of institutions or researchers	x		Types of institutions or researchers that might conduct research with biospecimens, and whether sharing of biospecimens might occur
2.6 Conditions and duration of storage	x	x	Conditions and duration of storage; the period of time that biospecimens may be stored, maintained, and used for research purposes
3. Items related to risk-benefit			
3.1 Risks and discomforts	x		Reasonably foreseeable risks or discomforts to the participant
3.2 Benefits	x		Reasonably expected benefits to the participant or to others
4. Consent part			
4.1 Consent documentation	x	x	Consent documentation for broad consent

### Language and readability analysis

The language used in the broad ICFs was also evaluated. Of the 54 broad ICFs reviewed, the English-language versions (*n* = 54) were analyzed using two validated readability metrics: the Flesch Reading Ease (FRE) and the Flesch–Kincaid Grade Level (FKGL). These metrics provide complementary information on reading ease and estimated educational level and were applied in accordance with established methods for assessing the readability of informed consent and clinical research materials [12, 16]. The FRE score ranges from 0 to 100, with higher values indicating easier comprehension [17]. According to standard interpretation guidelines, FRE scores of 91–100 indicate very easy text (approximately U.S. Grade 5 or 11-year-old reading level), 81–90 easy, 71–80 fairly easy, 61–70 standard (U.S. Grade 8–9 or 13–15-year-old reading level), 51–60 fairly difficult, 31–50 difficult, and 0–30 very difficult (college-level reading) [18]. The FKGL represents the estimated U.S. school grade level required for understanding [19]. For interpretation, FKGL scores were categorized as middle school (6 ≤ FKGL < 9), high school (9 ≤ FKGL < 12), and college (12 ≤ FKGL < 15). For the Thai-language versions (*n* = 54), as Thai is a scriptio continua language and lacks a validated readability metric equivalent to the FKGL that is appropriate for its linguistic structure, document length measured by word count using Microsoft Word’s built in segmentation was used as an objective proxy for information volume. For both language versions, word count and use of visual aids were also recorded for each element.

### Data analysis

Descriptive statistics were used to summarize the characteristics of the clinical trials and ICFs included in the analysis. The normality of continuous variables was assessed using the Shapiro-Wilk test. Continuous data were reported as means ± standard deviations (SD) or as medians with interquartile ranges (IQRs), as appropriate. Categorical variables were presented as frequencies and percentages. The proportion of broad ICFs that complied with each element was calculated to evaluate overall adherence to international standards. Spearman’s correlations were used to assess relationships among word count, readability (FRE), and reading complexity (FKGL) scores. To visualize trends in the data, a Pareto chart was created to highlight the most and least emphasized elements based on their relative contribution to document length. A Sankey diagram was also created using the “networkD3” package in R to illustrate the completeness distribution of each element across the documents. All analyses were conducted using Microsoft Excel and R software (Version 4.5.1).

## Results

### Characteristics of clinical trials associated with the included broad ICFs

The study documents comprised the total population of ICFs using a broad consent model during the study period. These documents were primarily derived from international clinical trials covering various therapeutic areas and patient populations. A total of 54 broad ICFs were included in our analysis. The main clinical trial characteristics are summarized in Table 2. Trial phases varied from Phase 1 to Phase 4, with Phase 3 being the most common (*n* = 36, 66.7%). Neoplasms were the most commonly studied condition (*n* = 24, 44.4%). Optional future research of biospecimens primarily focused on human genetics (*n* = 17, 31.5%), non-genetic biomarkers (*n* = 10, 18.5%), or both (*n* = 18, 33.3%), while some broad ICFs did not specify the type of future research (*n* = 9, 16.7%). Biospecimen sources included leftover samples (*n* = 26, 48.2%) and additional collections (*n* = 24, 44.4%), while some were from unclassified sources (*n* = 4, 7.4%).

**Table 2:** Clinical trial characteristics associated with the 54 broad ICFs analyzed in this study

Clinical trial characteristics	*n* (%)
Trial phase	
Phase 1	1 (1.9)
Phase 2	11 (20.4)
Phase 3	36 (66.7)
Phase 4	1 (1.9)
Others	5 (9.3)
Diseases/conditions being studied (according to ICD-10)	
Neoplasms	24 (44.4)
Certain infectious and parasitic diseases	9 (16.7)
Diseases of the blood and blood-forming organs and certain disorders involving the immune mechanism	8 (14.8)
Diseases of the genitourinary system	2 (3.7)
Diseases of the circulatory system	3 (5.6)
Diseases of the musculoskeletal system and connective tissue	3 (5.6)
Endocrine, nutritional and metabolic diseases	3 (5.6)
Diseases of the digestive system	1 (1.9)
Diseases of the eye and adnexa	1 (1.9)
None (i.e., healthy volunteers)	1 (1.9)
Type of optional future research	
Human genetics	17 (31.5)
Non-genetic biomarkers	10 (18.5)
Both genetic and non-genetic biomarkers	18 (33.3)
Unspecified	9 (16.7)
Sample collection for optional future research	
Leftover samples	26 (48.2)
Additional collection	24 (44.4)
Unclassified	4 (7.4)

*Abbreviations*: *ICF* informed consent form, *ICD-10* International Classification of Diseases, 10^th^ Revision

### Document length and content across broad ICF elements

The mean length of the English-language ICFs was 7.1 ± 2.8 pages (median, 7 pages; IQR, 3; range, 3–20 pages), whereas the Thai versions were generally longer, with a mean of 9.2 ± 3.8 pages (median, 8 pages; IQR, 3; range, 4–28 pages). Thai ICFs generally contained a higher word count across nearly all cases. The mean word count for the English documents was 2,188 ± 1,128 words (median, 1981 words; IQR, 475; range, 581–8688 words), while the Thai versions mean 3,022 ± 1,442 words (median, 2843 words; IQR, 746; range, 894–11344 words).

**Fig. 1:**
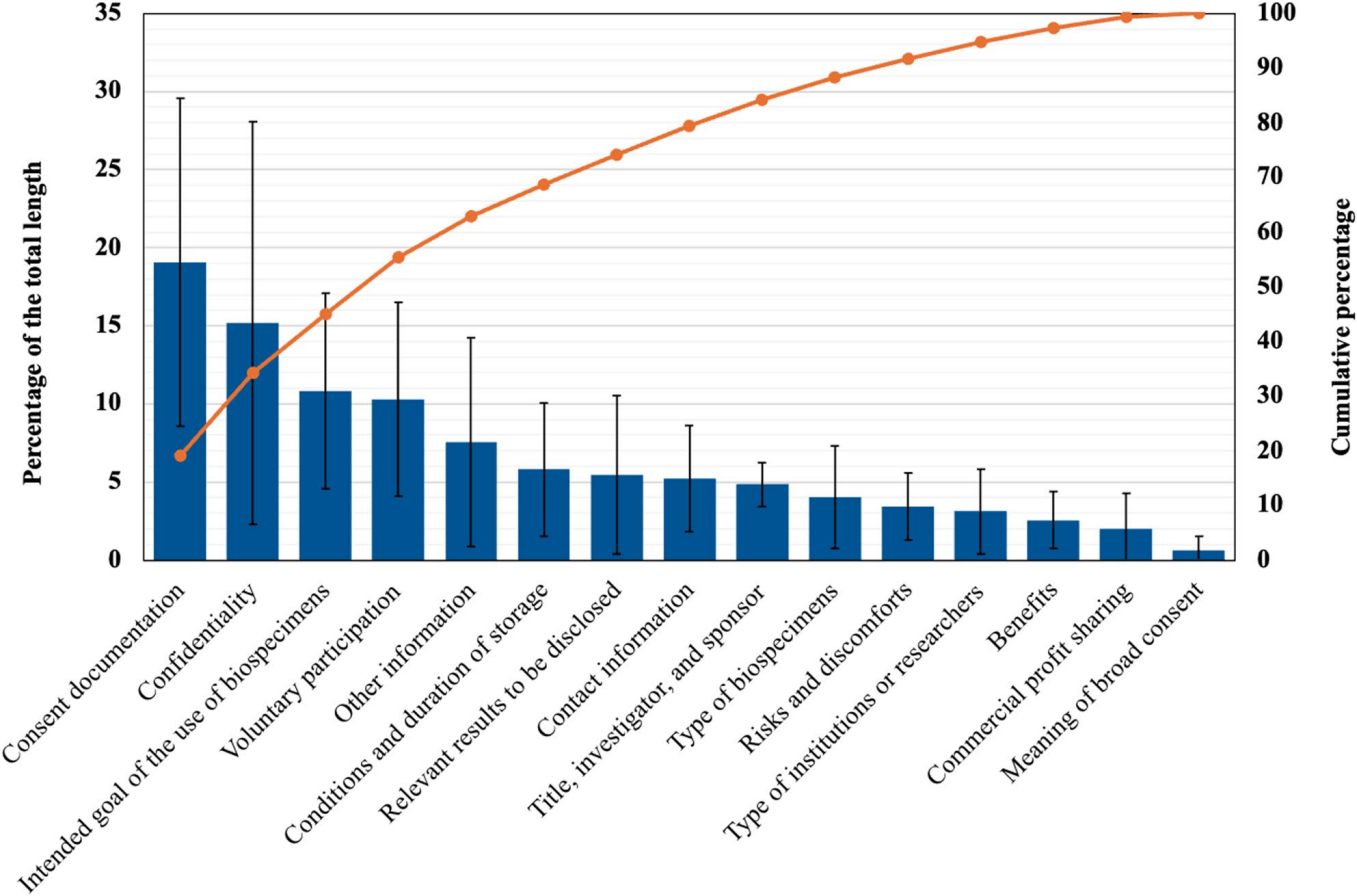
Distribution of the content length across essential elements of broad ICFs. The bar chart shows the average proportion of the total document length allocated to each of the 15 essential elements the broad ICFs. Error bars represent standard deviations. The orange line represents the cumulative percentage of total content

Figure 1 shows the average word count and proportion of document length allocated to each of the 15 elements. The analysis revealed that a small subset of elements accounts for the majority of the content. Four key components, including consent documentation, confidentiality, the intended goal of the use of biospecimens, and voluntary participation, together represented over half of the total length. Among these, consent documentation was the most detailed (19.1%), followed by confidentiality (15.2%), the intended goal of the use of biospecimens (10.8%), and voluntary participation (10.3%). The cumulative percentage line shows a sharp increase in the initial segments, indicating that these elements significantly shape the overall length of broad ICFs. Meanwhile, elements addressing benefits (2.6%), commercial profit sharing (2.0%), and the meaning of broad consent (0.6%) accounted for the smallest portions of text.

### Compliance of broad ICFs with ethical and regulatory requirements

**Fig. 2:**
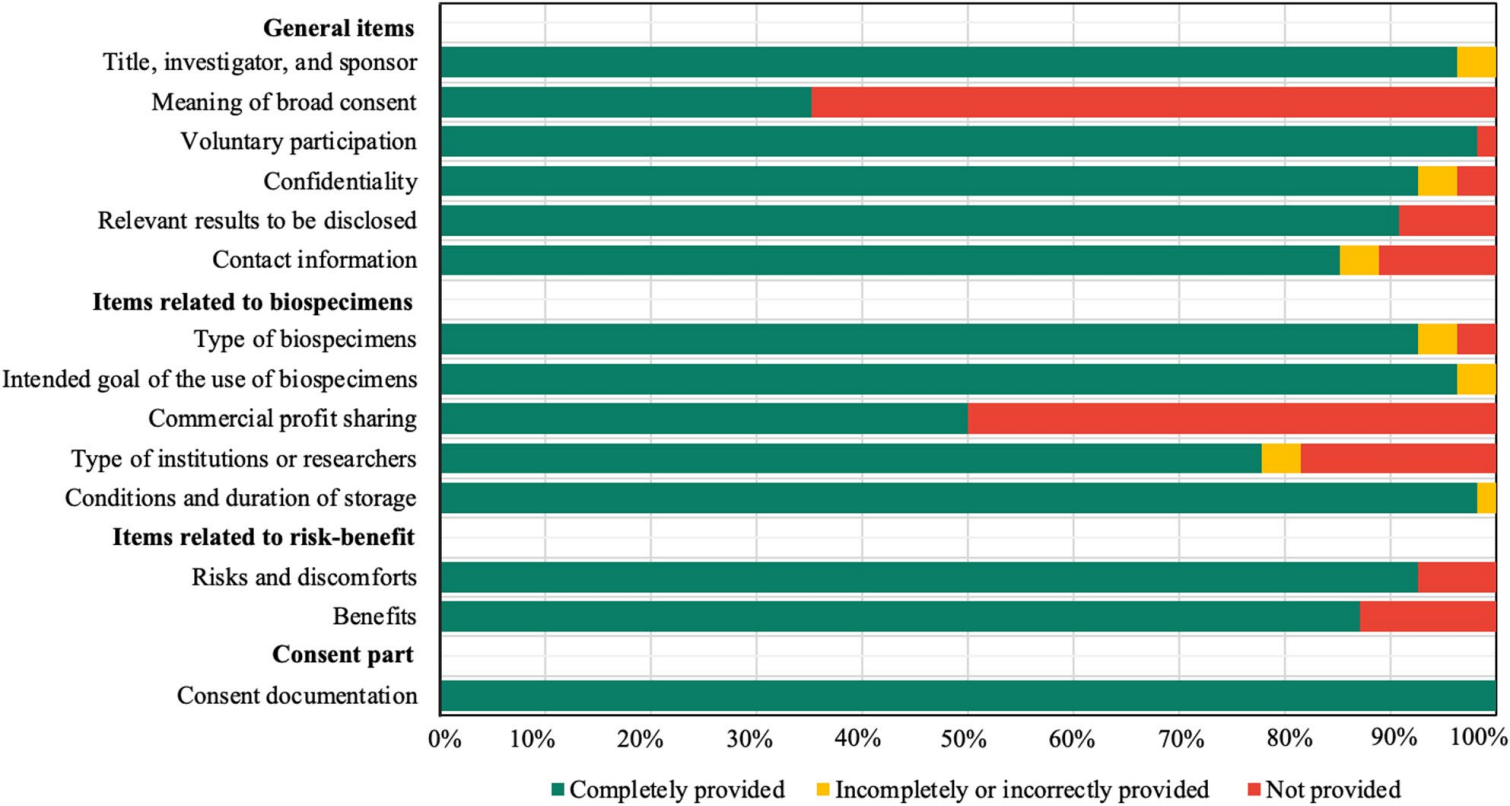
Compliance with 15 essential elements across broad ICFs. Each element is color-coded by the degree of compliance: completely provided (green), incompletely or incorrectly provided (yellow), and not provided (red)

Figure 2 presents the overall compliance of the 54 broad ICFs with 15 essential elements based on the 2018 Revised Common Rule and 2016 CIOMS Guidelines. Most of the essential elements were included in over 90% of the ICFs. The content provided in the original English broad ICFs was identical to that in the corresponding Thai versions across all included documents. The majority of broad ICFs provided complete information on consent documentation (*n* = 54, 100.0%), conditions and duration of storage (*n* = 53, 98.2%), and voluntary participation (*n* = 53, 98.2%). However, some elements such as the meaning of broad consent (*n* = 35, 64.8%) and commercial profit sharing (*n* = 28, 51.9%) were the most commonly missing information. Inadequate descriptions were frequently observed in elements such as contact information and confidentiality, where documents often directed participants back to the main study’s PICF rather than providing specific details within the standalone form. Descriptions of the meaning of broad consent were generally brief and often focused on the absence of future re-consent or re-notification for subsequent research use (e.g., “We will not contact you again for your permission…”). In contrast, statements addressing commercial profit sharing were typically longer, reflecting the need to explain issues related to intellectual property ownership and financial interests (e.g., “You and your heirs will not receive any financial or other benefits…”). The length and level of detail of these sections varied across documents, ranging from a few concise sentences to more detailed paragraphs, depending on document structure.

### Readability and language complexity of the included broad ICFs

**Fig. 3:**
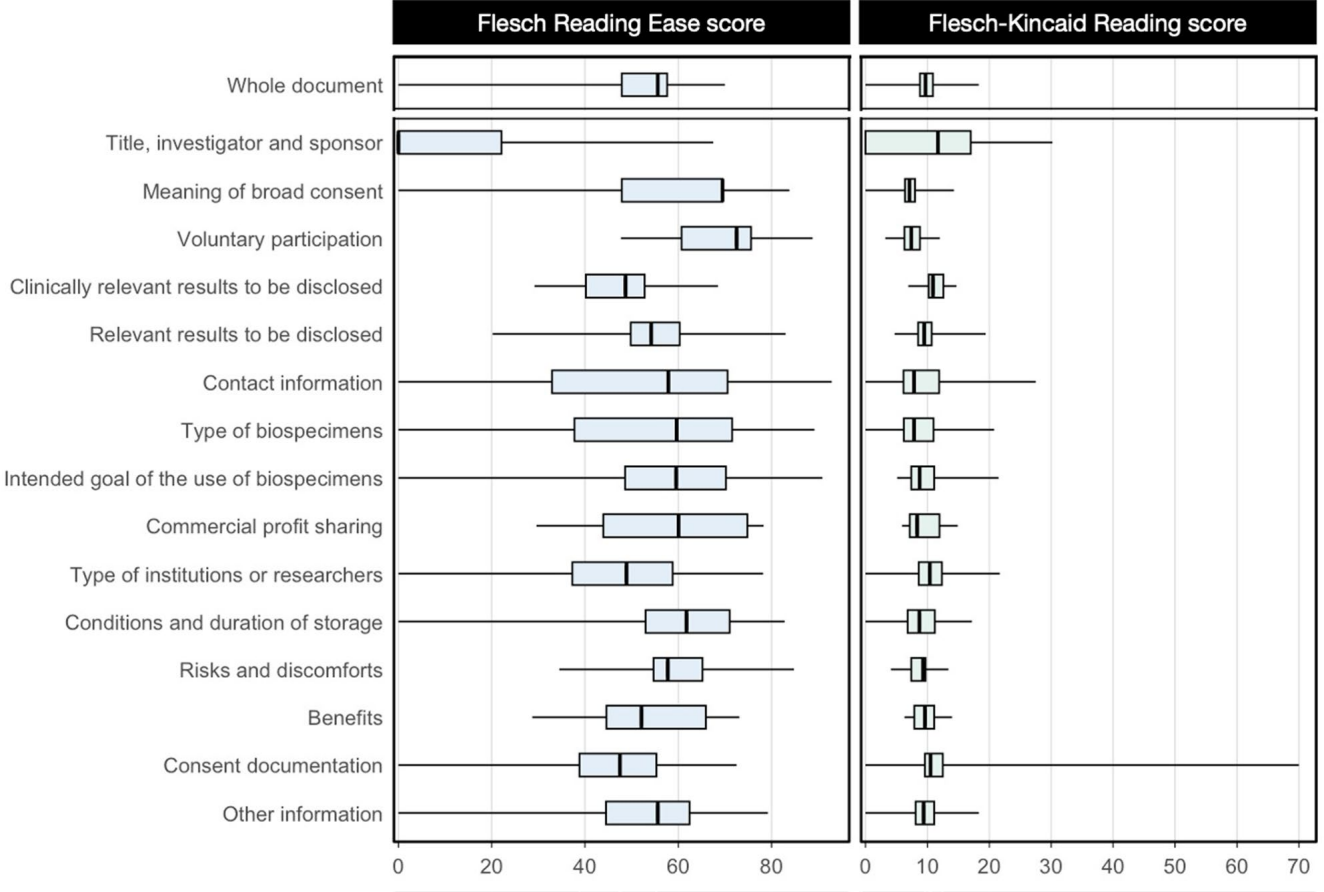
Median Flesch Reading Ease (FRE) and Flesch-Kincaid Grade Level (FKGL) scores across all included English-language broad ICFs. Boxplots display the median and interquartile range for FRE and FKGL for the entire document and for each of the 15 essential ethical and regulatory elements. Higher FRE scores indicate easier readability; higher FKGL scores indicate greater reading complexity

Regarding readability, the average FRE score of the English-language broad ICFs was 55.1 ± 7.0 (median, 55.6; IQR, 6.8; range, 39.1–71.5), indicating that most documents were fairly difficult to read (equivalent to high school to early college reading levels). The average FKGL score was 9.7 ± 1.5 (median, 9.7; IQR, 1.9; range, 6.5–13.1), corresponding to the 10th − 12th grade reading level in the educational system (Fig. 3). No significant correlation was observed between word counts and either FRE (ρ = -0.11, *p* = 0.686) or FKGL (ρ = 0.37, *p* = 0.173).

### Relationships and patterns of completeness and readability across broad ICF elements

**Fig. 4:**
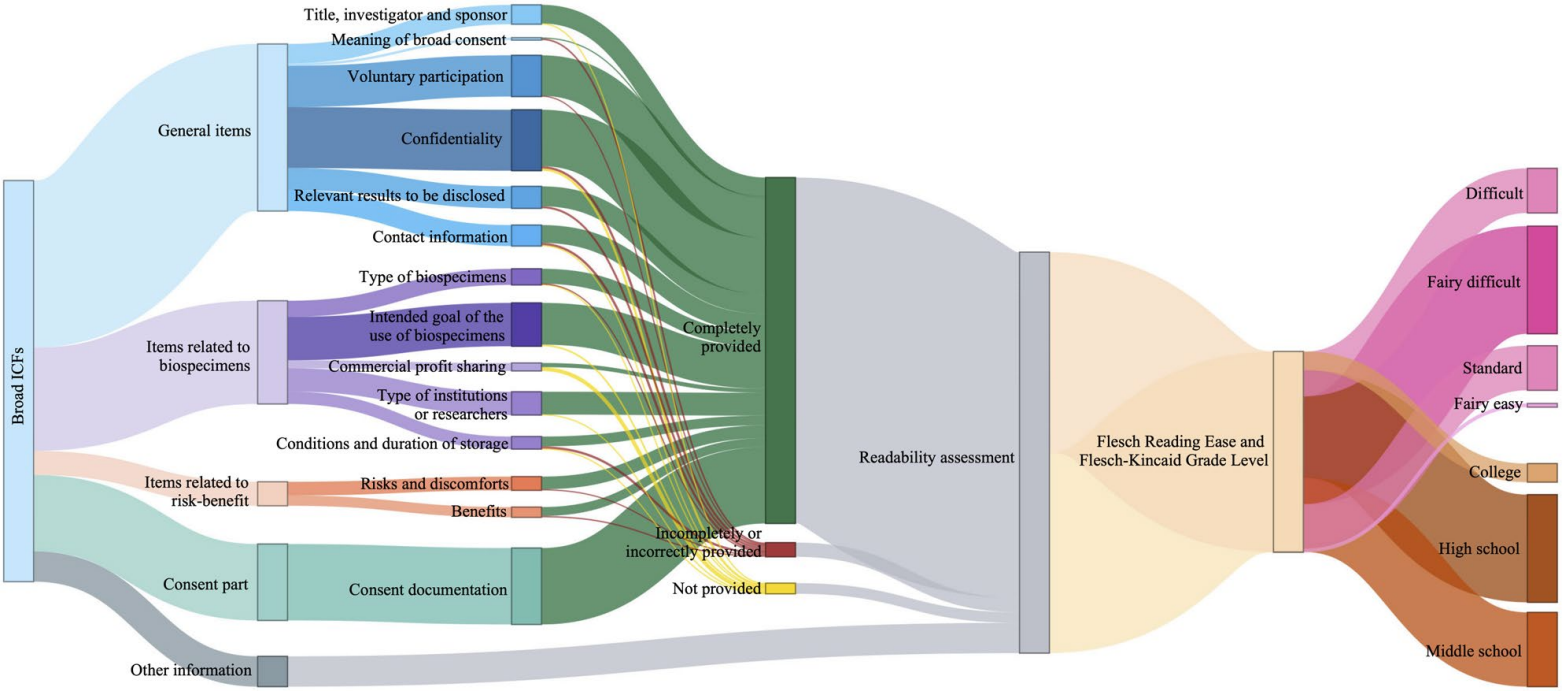
Sankey diagram summarizing the content length, regulatory compliance, and readability of the included broad ICFs. The diagram maps each of the 15 essential elements to their level of completeness (completely provided, incompletely or incorrectly provided, or not provided). Flow widths and color intensities represent the frequency of each category. The right side of the diagram links readability scores (FRE and FKGL) to estimated education levels, illustrating how essential elements vary not only in presence but also in accessibility

A visual summary of the relationship between the content of broad ICFs, their completeness, and readability is illustrated in a Sankey diagram (Fig. 4). This visualization highlights two key concerns: incomplete reporting of essential elements of broad consent and limited readability due to high linguistic complexity, both of which may compromise the ability of participants to make truly informed decisions.

In the underlying documents, several recurring patterns were observed. Certain elements were either comprehensively described or entirely omitted. Where information was incomplete, essential elements such as confidentiality and contact information often relied on cross-referencing the main study consent documents rather than providing standalone details within the broad ICF, whereas fully reported sections typically included detailed explanations of participant rights, procedures, and implications.

## Discussion

This empirical study provides a systematic assessment of broad ICFs used in international multicenter clinical trials conducted after the implementation of the 2018 Revised Common Rule. Our findings revealed both strengths and persistent gaps in how ethical and regulatory requirements are operationalized in research practice, particularly within an international multicenter clinical trial context in Thailand. While foundational elements, such as voluntary participation, confidentiality, and consent documentation were generally well addressed, some key biospecimen-specific issues, such as the meaning of broad consent and commercial profit sharing, were sometimes omitted or insufficiently addressed. These findings are consistent with concerns raised in prior literature regarding the ambiguous or incomplete nature of broad consent [6, 7, 20]. Failure to provide such information may compromise participants’ informed decisions, especially in contexts where the scope of secondary research is undefined at the time of consent and may render consent invalid.

One of the most significant gaps was the failure to clearly explain the meaning of broad consent. Despite the requirements of the 2018 Revised Common Rule, more than 60% of broad ICFs in this study did not clarify that participants would not be informed about future uses of their biospecimens if such research falls within the scope of broad consent. Prior research has shown that participants often assume they will be re-contacted or retain control over future uses of their biospecimens unless explicitly informed otherwise [21, 22]. Such gaps constitute ethical challenges and may also compromise public trust, particularly if participants later discover unanticipated uses of their biospecimens [23].

Another commonly missing element in broad ICFs was the disclosure of commercial profit sharing, if any, arising from the use of biospecimens. This finding aligns with broader ethical concerns regarding the commercialization of genomic discovery, especially in contexts where sensitivities surrounding exploitation and benefit-sharing are pronounced [24]. For instance, the H3Africa Consortium reported that fewer than half of broad ICFs mentioned commercialization, and only a few addressed profit-sharing with participants [25]. Participants, particularly those in vulnerable settings, may feel exploited if they later learn that their biospecimens were used in profit-driven research without their awareness [22]. This finding underscores the need for transparent and ethically robust framework for communicating the potential commercialization and benefit-sharing implications of research involving biospecimens to participants [20].

The analysis also identified challenges related to document length and readability. Many broad ICFs were lengthy, which may reflect cultural tendencies toward providing thorough explanations [26]. However, excessive information within these forms may have obscured key information, potentially affecting participants’ understanding [16]. Linguistic complexity further compounded this issue. According to plain language guidance and recommendations, ICFs should be written at a 6th − 8th grade reading level to support comprehension across diverse populations [27–29]. However, the average FKGL score of the broad ICFs analyzed in this study exceeded 9.7, placing most documents above the recommended reading level for communication.

To address these deficiencies, the adoption of standard ICF templates should be encouraged. For instance, the SACHRP template provides clear guidance on how to address various participant responses to broad consent [8]. Such tools can serve as both checklists and practical guides for improving ethical coverage and language accessibility. However, standardization alone is not sufficient. Broad ICFs should also give careful attention to how information is conveyed—using plain language, avoiding legal or scientific jargon, and ensuring that ethically sensitive topics are presented clearly and respectfully, in a concise manner [30]. As emphasized in the CIOMS guidelines and other ethical frameworks, truly informed consent requires not only regulatory compliance but also clarity, accessibility, and respect for participant autonomy.

This study has some limitations. First, the scope of data was limited to broad ICFs from international multicenter clinical trials that were accessible for review, which may not represent the full spectrum of research practice across other contexts or study designs. Second, the evaluation relied on document analysis, and the readability assessment was based on quantitative metrics. While readability metrics (namely FRE and FKGL) provide useful benchmarks, they do not capture other critical aspects of comprehension, such as formatting, layout, and health literacy. The readability assessment did not specify lay readers as the target audience, which may affect interpretations of understandability. Moreover, cultural factors and language preferences, such as the tendency toward elaboration for clarity, should also be considered [18, 26]. Third, the study did not incorporate perspectives from REC members, researchers, or participants, which would be necessary to provide a more comprehensive understanding of how broad ICFs function in research practice.

## Conclusion

This study provides insights into the ethical and regulatory characteristics of broad ICFs used in international multicenter clinical trials involving biospecimens. Overall, most ICFs were found to comply with key regulatory and ethical requirements. However, variation in content completeness was observed, with some forms lacking clear explanations of broad consent or information related to commercial profit sharing. In addition, the considerable length and limited readability of several documents may pose challenges for participant comprehension. By systematically examining the actual content and readability of broad ICFs, rather than stakeholder perspectives, this study addresses an important gap in the existing literature, particularly in the context of international multicenter research conducted in Thailand. Taken together, these findings highlight opportunities to improve the clarity, conciseness, and accessibility of broad ICFs. Greater use of shared templates and careful consideration during ethics review may help support participant understanding and protection in research involving the long-term storage and future use of biospecimens.

## Data Availability

The datasets included and analyzed in this study are available from the corresponding author upon request.
